# Turnover and activity-dependent transcriptional control of NompC in the *Drosophila* ear

**DOI:** 10.1016/j.isci.2021.102486

**Published:** 2021-04-29

**Authors:** Nicholas Boyd-Gibbins, Camille H. Tardieu, Modesta Blunskyte, Nerissa Kirkwood, Jason Somers, Joerg T. Albert

**Affiliations:** 1 Ear Institute, University College London, 332 Gray’s Inn Road, London WC1X 8EE, UK; 2 The Francis Crick Institute, 1 Midland Road, London NW1 1AT, UK; 3 Centre for Mathematics and Physics in the Life Sciences and Experimental Biology (CoMPLEX), University College London, Gower Street, London WC1E 6BT, UK; 4 Department of Cell and Developmental Biology, University College London, Gower Street, London WC1E 6DE, UK

**Keywords:** Biological sciences, Molecular biology, Sensory neuroscience

## Abstract

Across their lives, biological sensors maintain near-constant functional outputs despite countless exogenous and endogenous perturbations. This sensory homeostasis is the product of multiple dynamic equilibria, the breakdown of which contributes to age-related decline. The mechanisms of homeostatic maintenance, however, are still poorly understood. The ears of vertebrates and insects are characterized by exquisite sensitivities but also by marked functional vulnerabilities. Being under the permanent load of thermal and acoustic noise, auditory transducer channels exemplify the homeostatic challenge. We show that (1) NompC-dependent mechanotransducers in the ear of the fruit fly *Drosophila melanogaster* undergo continual replacement with estimated turnover times of 9.1 hr; (2) a *de novo* synthesis of NompC can restore transducer function in the adult ears of congenitally hearing-impaired flies; (3) key components of the auditory transduction chain, including NompC, are under activity-dependent transcriptional control, likely forming a transducer-operated mechanosensory gain control system that extends beyond hearing organs.

## Highlights

• *De novo* NompC synthesis restores auditory transduction in congenitally deafened flies.
• Complete turnover of NompC mechanotransducers within less than 24 hr.
• Activity-dependent transcriptional control of transducers controls auditory function.

## Graphical abstract

**Figure:**
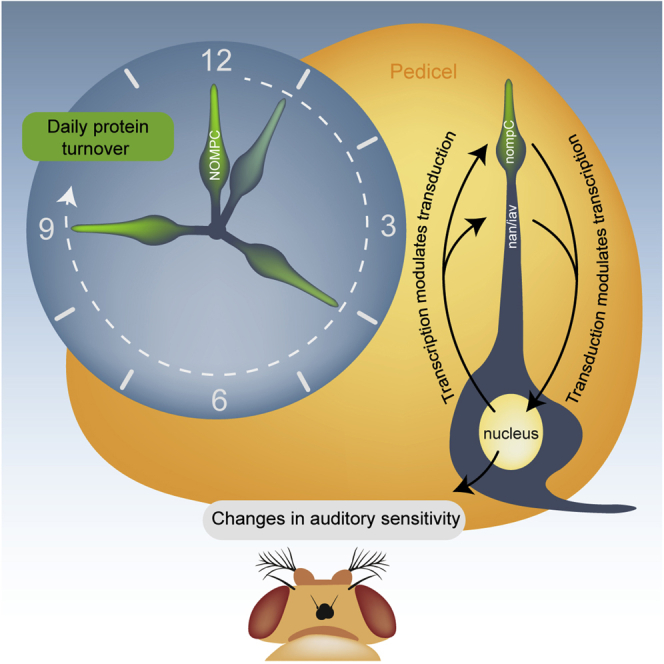

## Introduction

Ever since the seminal work of Rudolf Schoenheimer (Schoenheimer, 1946), the predominant theory of life is based on the concept of dynamic equilibria, where seemingly invariable states–or performances–are in truth the product of a homeostatic balance between assembling and disassembling processes. Questions around the molecular and mechanistic logic of homeostasis have remained at the forefront of the life sciences; their answers will also be of relevance for understanding the process of aging.

During their development and life courses, biological tissues are being constantly modeled and re-modeled. Here, the particular roles of mechanical forces in shaping developmental (Eder et al., 2017; Petridou et al., 2017) or homeostatic processes–such as adaptive bone remodeling (Rubin et al., 2006)–are becoming increasingly recognized. Examples of intra- or intercellular mechanical feedback systems include interactions with the actin cytoskeleton (Blanchoin et al., 2014) or integrin-mediated mechanotransduction (Sun et al., 2016).

The transduction of mechanical forces into biochemical signals is thus a key requirement for the development and homeostatic maintenance of all complex organs. Those organs that are themselves specialized for the transduction of minute mechanical forces, such as hearing organs, are arguably among the most complex sensory organs that have evolved (Albert and Kozlov, 2016). The act of hearing starts with the activation of auditory transducer complexes (ATCs). ATCs are formed by membrane-bound, and force-gated, mechanotransducer channels (METs), which are linked to cytoskeletal structures and interact with various motor proteins that provide adaptation and amplification (Qiu and Müller, 2018). Auditory METs (aMETs) respond to nanometer displacements of their receiver structures (such as stereociliary hair bundles in vertebrate hair cells or antennal sound receivers in Dipteran insects (Albert and Kozlov, 2016)); but, despite the documented presence of proteostasis network (PN) components (Bokolia and Mishra, 2015) required for MET localization (Lee et al., 2008; Park et al., 2013, 2015) and the knowledge that larger (Dice et al., 1979) or mechanically loaded (Kjaer et al., 2006) proteins tend to have higher turnover rates, the protein dynamics (and proteostasis) of aMETs is almost entirely unknown. The study of auditory transducer proteostasis is of scientific importance for two reasons: aMETs are (1) the most sensitive type of mechanotransducers, which are, even in the absence of audible sound, constantly flickering between open and closed states, merely responding to the gating forces provided by thermal noise (Hudspeth et al., 2000); (2) they are part of a sensory system that shows substantial age- and noise-dependent vulnerability in both humans (Liberman, 2017; Gates and Mills, 2005) and *Drosophila* (Christie et al., 2013; Keder et al., 2020). In fact, proteostasis has been recognized as a major factor in aging processes (Taylor and Dillin, 2011; Toyama and Hetzer, 2013). Advancing our knowledge of auditory transducer homeostasis thus has also the potential to guide the way to novel protective interventions in humans.

Previous studies have found that membrane proteins show higher turnover rates than synaptic or mitochondrial ones (Dörrbaum et al., 2018) and some key molecular players for membrane protein turnover, such as ubiquitin, have been identified (MacGurn et al., 2012).

Protein dynamics of both cilia (the cellular sites of mechanotransduction in insect auditory neurons) (Mirvis et al., 2018; Hsu et al., 2017) and ‘stereocilia’ (the cellular sites of mechanotransduction in vertebrate auditory hair cells) (Zhang et al., 2012; Grati et al., 2006) have been studied and it has been suggested that ion channels are characterized by particularly high turnover rates; but very little is known about MET channel turnover. This is largely due to the fact that the molecular identification of aMETs has proven a scientific challenge (Qiu and Müller, 2018). At present, candidate transducer channels have been identified for both mammals (Qiu and Müller, 2018) and insects (Albert and Göpfert, 2015). In *Drosophila*, it was shown that the transient receptor potential (TRP) channel NompC (=TRPN1) is (1) essential for sound receptor function (Effertz et al., 2011), (2) required for the mechanical integrity (gating springs) of auditory transducers (Effertz et al., 2012; Zhang et al., 2015), and (3) can form a *bona fide* mechanotransducer itself (Yan et al., 2013). NompC thus meets the key criteria for being a *Drosophila* auditory transducer channel. Another essential component of fly hearing is the heterodimeric channel formed by Nanchung (Nan) and Inactive (Iav), which is unique to chordotonal organs (ChOs) (Kavlie and Albert, 2013). The Nan/Iav channel is required for the generation of sound-evoked compound action potential (CAP) responses from the *Drosophila* ear. The current model of *Drosophila* hearing regards NompC as the auditory transducer channel proper, with Nan/Iav acting as a downstream amplifier, or modifier, channel (Albert and Göpfert, 2015).

We used NompC to study protein dynamics and transcriptional control of auditory transducers in Johnston's organ, a large chordotonal organ (Kavlie and Albert, 2013), which forms the *Drosophila* antennal ear. Our results show that NompC-dependent transducers undergo a continual turnover with an estimated turnover time (∼76% completion) of <10 hr. The *de novo* synthesis of NompC can restore auditory transducer function in congenitally hearing-impaired flies and an activity-dependent transcriptional control of NompC expression acts to re-balance important system properties (e.g., the nonlinearity, frequency selectivity, and amplification of the antennal sound receiver). Transducer turnover and dynamics are likely to form a key mechanism of *Drosophila* auditory homeostasis.

The *Drosophila* auditory system forms a powerful model to study and quantify the general act of hearing (Albert and Kozlov, 2016; Albert et al., 2020), and specifically the elementary process of auditory transduction (Albert et al., 2007a). As a direct result of a mechanical gating, force transmission between the fly's auditory transducer channels and their external sound receiver is inherently reciprocal. Transducer gating thus introduces characteristic mechanical signatures, e.g., gating compliances (Albert et al., 2007b; Howard and Hudspeth, 1988), into the receiver's mechanics. These circumstances allow for assessing auditory transducer function (or malfunction) in the intact ears of live flies. Partly as a result of their unique mechanical coupling to extra- and intracellular components (which hampers the heterologous analysis of candidate channel proteins), the molecular identification of auditory transducers has proven challenging (Corey, 2006; Fettiplace, 2016; Albert and Göpfert, 2015). At the time of writing, only few molecular candidates, which meet the key criteria for pore-forming subunits of true auditory transducer channels have emerged in both insects and vertebrates; these are the two transmembrane channel-like (TMC) proteins TMC1 and TMC2 in vertebrates and the transient receptor potential (TRP) channel NompC (=TRPN1) in insects. We here concentrate on the insect channel NompC. The ears of NompC-deficient (*nompC*^*null*^) flies have been reported to lose the electrical responses to sound (Effertz et al., 2011), as well as the receiver's characteristic nonlinear compliances (=stiffness drops) associated with the gating of sensitive auditory transducer channels (see ref(Effertz et al., 2012)).

## Results

### An adult-specific rescue of congenital deafness

**Figure 1:**
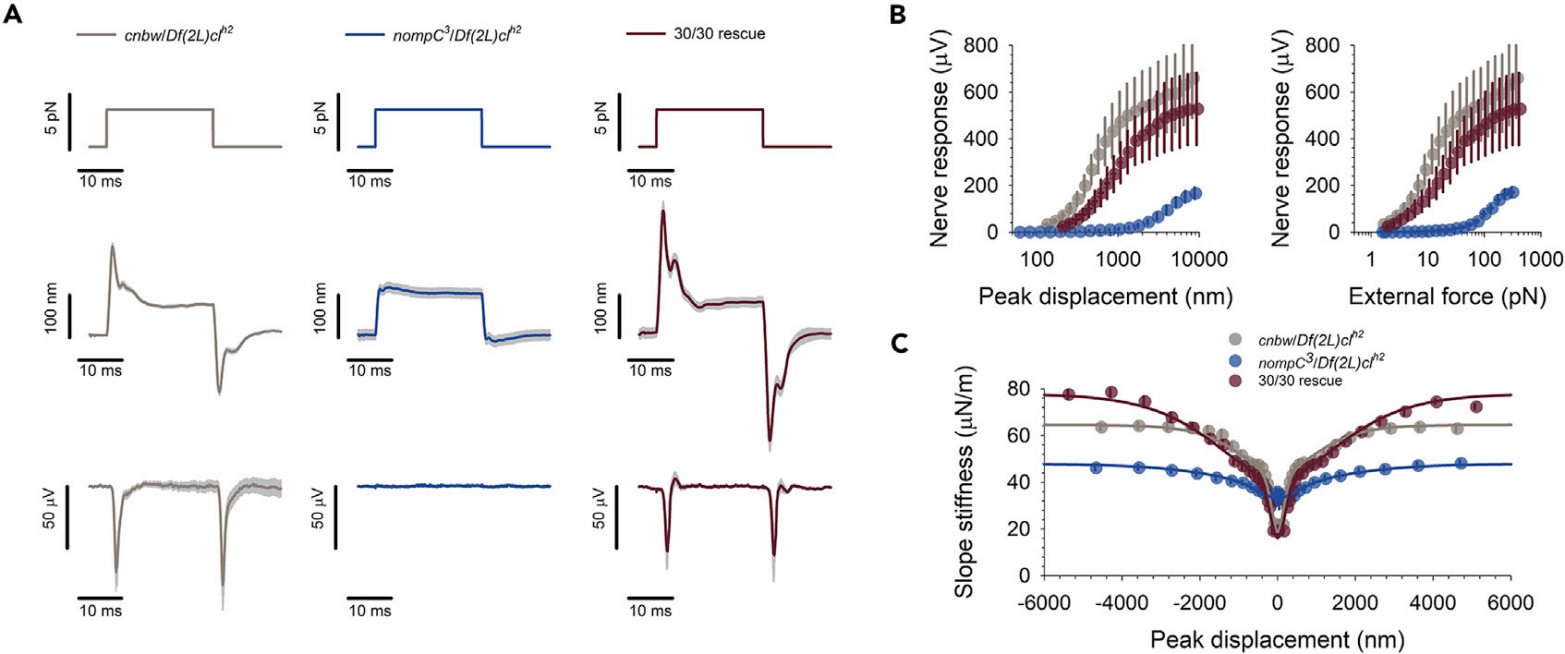
NompC-L-GFP fusion protein restores auditory transduction to *nompC*^*null*^ mutant background (A) Mechanical and electrical responses to force step actuation of the *Drosophila* antennal ear in *cnbw* control flies (left, grey, N = 6), nompC^3^ mutants (middle, blue, N = 5) and 30/30 rescue flies (right, brown, N = 7); top traces: force steps, middle traces: antennal displacement, bottom traces: compound action potential (CAP) responses. Shaded areas show standard errors of the median. (B) Magnitude of CAP responses as a function of antennal peak displacement (left) and size of the force step (right). (C) Slope stiffness of the antennal receiver as a function of peak displacement. Lines show fits of a two transducer-type gating spring model. Error bars are standard errors of the median.

We were first interested to see if NompC–supplied to congenitally NompC-deficient (*nompC*^*3*^) flies upon adulthood (i.e., upon the flies' eclosion from their pupae)–could restore auditory transducer function. To this end, we generated a fly line that carried (1) a NompC-Gal4 driver, (@) a temperature dependent suppressor of Gal4 activity (tub-Gal80^ts^) together with (3) a UAS-NompC-L-GFP rescue construct in a *nompC*^*3*^ mutant background (henceforth simply referred to as *experimental flies*; see Method section for complete genotypes). In experimental flies, NompC expression would be suppressed at an ambient temperature of 18°C but expression would be initiated once flies are transferred to 30°C, thus allowing for probing an adult-onset supply of NompC in a NompC-deficient background. As a first positive control, and to test the functionality of the NompC-L-GFP rescue construct, we compared *nompC*^*null*^ flies to their *cnbw* control strain and to experimental flies that were raised and kept at 30°C (30/30 rescue) (Figure 1). We expected the 30/30 rescues to have uninhibited, control-like NompC supply. Consistent with previous findings (Effertz et al., 2012), the ears of our *nompC*^*null*^ fly strain lose the characteristic displacement overshoot (Figure 1A, middle), nerve responses (Figure 1B), and gating compliances (Figure 1C, blue) seen after force step actuation. The ears of 30/30 rescues, however, display all mechanical and electrical signatures of auditory transducer gating and closely resemble those of *cnbw* control flies (Figures 1A–1C). The NompC-L-GFP fusion protein employed in this study thus forms a fully functional transducer channel.

We then wanted to know if *de novo* synthesized NompC - supplied post-developmentally after the flies' eclosion from their pupae - could still be incorporated into the functional transducer complexes. This would indicate the existence of dedicated transport machinery, a crucial prerequisite for a potential homeostatic regulation of active transducer channel numbers in the adult fly ear. We compared the ears of experimental flies under two rearing conditions: (1) Flies that were raised and kept at 18°C, in which NompC expression should be continually blocked (18/18 continued block) and (2) flies that were raised at 18°C but moved to 30°C upon eclosion, which should block the expression of NompC during JO development but initiate its *de novo* synthesis in adults (18/30 adult release flies).

**Figure 2:**
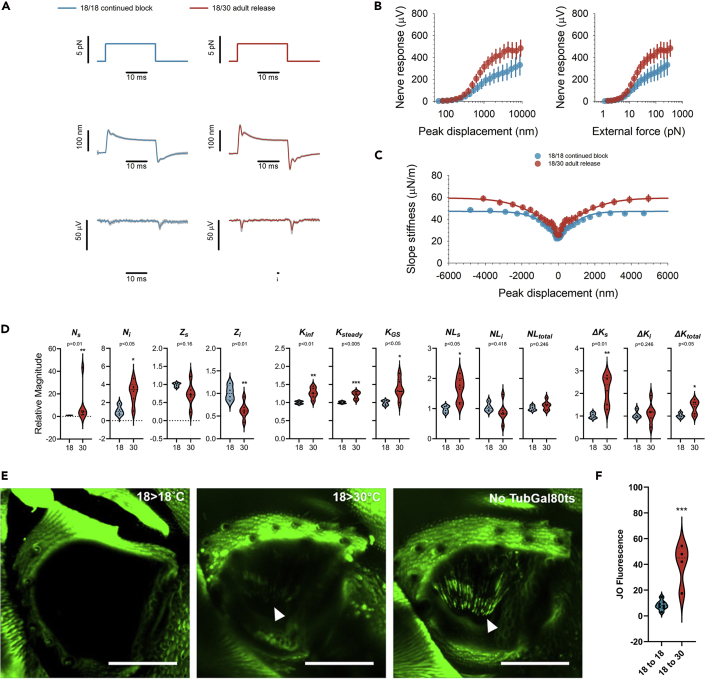
Adult-specific expression of NompC-L-GFP fusion protein restores key parameters of auditory transducer function to *nompC*-deficient flies (A) Mechanical and electrical responses to force step actuation of the *Drosophila* antennal ear in flies raised and kept at 18°C (18/18 continued block) (left, gray, N = 5) and flies raised at 18°C and transferred to 30°C upon eclosion (18/30 adult release) (right, brown, N = 7); top traces: force steps, middle traces: antennal displacement, bottom traces: compound action potential (CAP) responses. Shaded areas show standard errors of the median. (B) Magnitude of CAP responses as a function of antennal peak displacement (left) and size of the force step (right). (C) Slope stiffness of the antennal receiver as a function of peak displacement. Lines show fits of a two transducer-type gating spring model. Error bars are standard errors of the median. (D) Key parameters of auditory transducer function as resulting from the fits in (C). All parameters are expressed in relative terms (divided by their respective values in the 18/18 controls). Parameters (see methods and ref(Effertz et al., 2012)): number of sensitive (*N*_*s*_) and insensitive (*N*_*i*_) transducer channels; sensitive (*z*_*s*_) and insensitive (*z*_*i*_) single channel gating forces; asymptotic stiffness (*K*_*inf*_); parallel stiffness (*K*_*steady*_); gating spring stiffness (*K*_*GS*_); extent of nonlinearity of sensitive (*NL*_*s*_) and insensitive (*NL*_*i*_) transducers and both combined (*NLtotal*); stiffness relief for sensitive (*ΔK*_*s*_) and insensitive (*ΔK*_*i*_) transducers and both combined (*ΔK*_*total*_). (E) (right) Without a Gal80^ts^ mediated block, NompC-L-GFP fluorescence is clearly visible in JO neurons and the tips of their ciliary dendrites (arrow); (left) 18/18 flies, in which NompC-L-GFP expression is blocked, lack fluorescence in JO; (middle) after an adult specific release of the Gal80^ts^ mediated block, JO neurons of 18/30 flies show clear ciliary fluorescence (arrow). Scale bars: 50 μm. (F) Quantitative comparison of JO fluorescence in 18/18 (N = 7) and 18/30 (N = 4) flies.[For all panels: parametric or non-parametric significance tests were selected after prior checks for normality and equal variance. All indicated significances, or non-significances, remain valid (threshold p<0.05) when using non-parametric tests].

**Figure 3:**
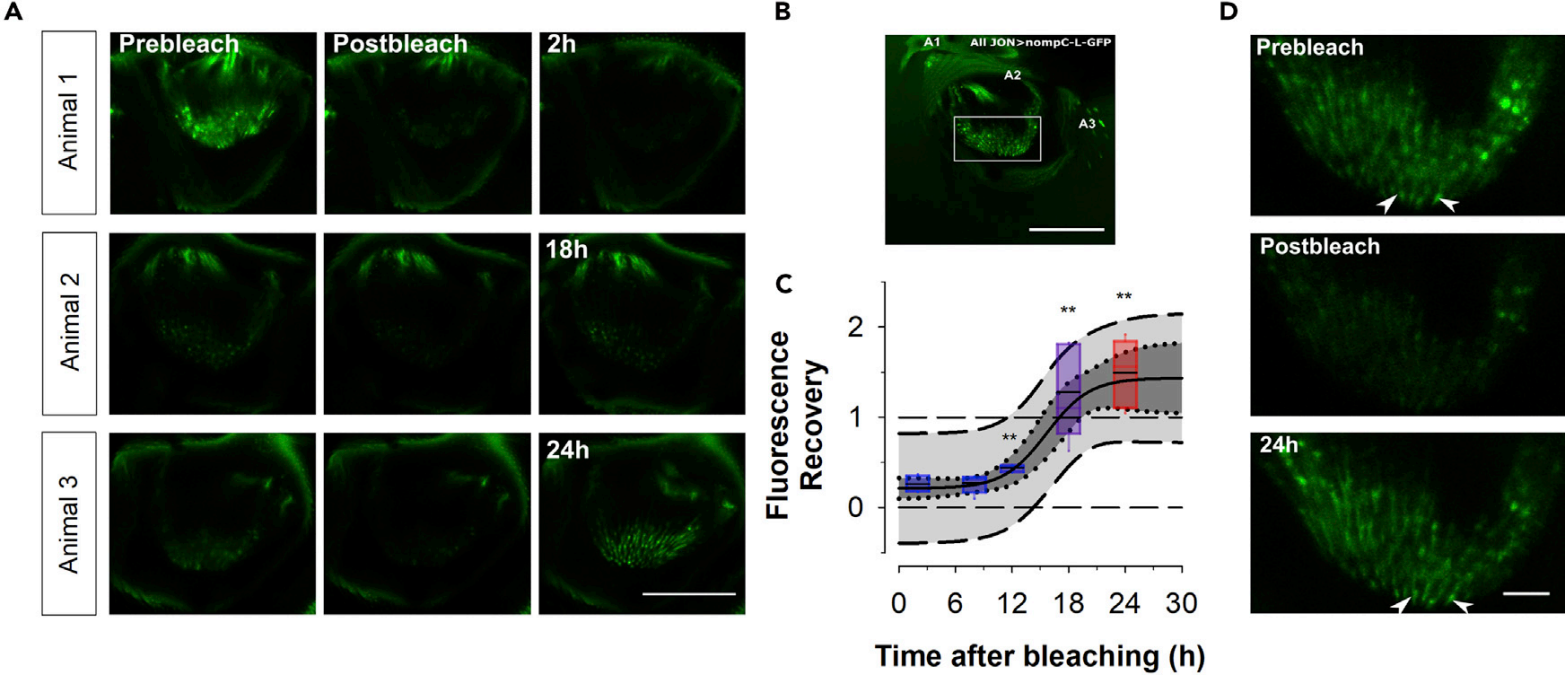
Fluorescent recovery after photobleaching of NompC-L-GFP (A) Confocal images showing pre-bleach, post-bleach and post-bleach after 2 hr (top row), 18 hr (middle row) and 24 hr (bottom row). Each row shows images from an individual animal. Scale bar: 50 μm. (B) Z-projections of confocal images of the JO of flies expressing the rescue construct NompC-L-GFP driven by NompC-Gal4 in a null *nompC*^*3*^ background. The white box surrounds the distal cilia tips and shows the area targeted for photobleaching. (C) Fluorescent recovery calculated from intensity values in the bleached area at different timepoints after bleaching. Boxes represent 5th and 95th percentile with the horizontal bar representing median values. Fit parameters: time constant (τ) = 2.29hr[corresponding to a ~76% (=4τ) turnover time of 9.16hr]; initial relative fluorescence, *F*_*start*_ = 0.212; final relative fluorescence, *F*_*end*_ = 1.435; midpoint *t*_*0.5*_ = 15.67hr[see Figures S3 and S4 for further details]. (D) Confocal images of the ~66 × 44 μm target areas for pre-bleach (top), post-bleach (middle) and after 24 hr (bottom). Arrowheads point to the distal cilia tips. Scale bar: 10 μm.

Comparing the two conditions reveals significant increases in all parameters of auditory transducer function in 18/30 adult release flies (Figure 2): (1) Their initial displacement overshoot after force step actuation is enhanced (Figure 2A), (2) their nerve responses are larger (Figure 2A, bottom; Figure 2B), and (3) their nonlinear gating compliances are more pronounced (Figure 2C). A detailed quantitative analysis (Figure 2D) shows that the changes also include a rise in the number of predicted transducer channels. Both sensitive transducers, *N*_*s*_ (p<0.01; ttest), and insensitive transducers, *N*_*i,*_ (p<0.05; ttest), increase significantly in 18/30 adult flies. Whereas the drop observed in single channel gating forces failed to reach significance for the sensitive transducers, *z*_*s*_ (p = 0.16; ttest), it was significantly reduced in the insensitive transducer population, *z*_*i*_ (p<0.01; ttest). Also other key parameters of transducer mechanics, such as the asymptotic stiffness, *K*_*inf*_ (p<0.01; ttest), the receiver's steady-state stiffness, *K*_*steady*_ (p<0.05; ttest), the total gating spring stiffness, *K*_*GS*_ (p<0.05; ttest), and the extent of nonlinearity of the sensitive transducer population, *NL*_*s*_ (p<0.01; ttest), showed significant increases. The extent of nonlinearity of insensitive transducers, *NL*_*i*_ (p = 0.418; ttest), however, and the receiver's total extent of nonlinearity, *NL*_*total*_ (p = 0.246; ttest), remained constant. A parameter specific to transducer function, and independent of passive receiver mechanics, is the absolute stiffness relief, Δ*K*, which the gating of transducers provides to the receiver. The total Δ*K* (Δ*K*_*total*_, p<0.05; ttest) rose significantly, but this increase was carried exclusively by the sensitive transducers, Δ*K*_*s*_ (p<0.01; ttest), whereas the stiffness relief for insensitive transducers remained stable, Δ*K*_*i*_ (p = 0.246; ttest). Taken together, these changes are a clear indication that novel mechanotransducers have been integrated into the ciliary transduction zones of JO neurons. The rescue seen in the 18/30 flies happened on top of a partial rescue that could already be observed in the 18/18 controls. 18/18 flies thus were, in effect, not completely deaf (NompC-null) flies but rather hard-of hearing (NompC-impaired) flies. This is either the result of an occasionally reported ‘leakiness’ of the UAS rescue construct or an incomplete Gal4 suppression by Gal80^ts^ (Port et al., 2020; Hudson and Cooley, 2014), which–especially for functional systems operating with very low protein numbers, such as the *Drosophila* auditory transducer machinery–can already lead to a non-specific, partial rescue (Kavlie et al., 2010). As a complete suppression of transducer function was not required for our study, we did not explore these issues further.

But as we used a temperature-controlled gene delivery paradigm for a poikilothermic–i.e., temperature-unstable–animal, the question arises if some of the observed changes reflect a direct temperature effect on auditory transduction. Previous studies have found robust temperature compensation mechanisms in insect auditory receptors (Roemschied et al., 2014), but data on the effects on auditory transduction proper are still lacking. We therefore conducted a temperature control experiment in wild-type flies (Oregon-R). We found that a temperature increase from 18°C to 30°C had indeed significant (albeit small) effects on some transducer parameters (Figure S1). All of these, however, changed the respective transducer parameters in the opposite direction as compared to the 18/30 rescue flies: Transducer numbers (*N*_*s*_ and *N*_*i*_), for example, showed a (non-significant) tendency to decrease at 30°C, whereas they significantly increased in the 18/30 rescue flies; the stiffness relief provided by the gating of (sensitive) auditory transducers (*ΔK*_*S*_) also showed a significant decrease in 30°C wild-type control flies but significantly increased in 18/30 rescue flies. The *de novo* expression of NompC, thus, not only had to rescue the functional deficits of the genetic background but also a temperature-accelerated transducer aging. These findings further validate the observed recovery.

To probe and visualize this integration more directly, we exploited the fact that our NompC rescue construct was fused to a GFP reporter. While flies raised under a continued Gal80^ts^-mediated block of NompC expression (18/18 continued block) showed close-to-zero levels of specific fluorescence in JO (Figure 2E, left), fluorescence was restored to the apical cilia of JO neurons in 18/30 adult release flies (white arrow head in Figure 2E, middle) and showed a significant increase in fluorescent intensity at the ciliary tips (from median 7.87 to 44.97, p = 0.0005; ttest) compared to 18/18 flies (Figure 2F).

### NompC transducers undergo continual turnover

The suggestion that newly expressed NompC keeps being transported, and functionally integrated, into the mechanotransduction sites of JO neurons opens the possibility that an *in vivo* homeostatic maintenance machinery exists, which employs transducer channel turnover to regulate JO sensitivity. To test this idea, we once again made use of the fluorescent nature of our rescue strategy. Using fluorescence recovery after photobleaching (FRAP), we quantified the turnover of NompC in the JOs of adult flies (Figure 3). FRAP analysis indeed confirmed a continuous turnover of NompC channels. While no recovery was observed 2 hr post-bleaching, fluorescence intensities rose steadily thereafter and, at 24 hr, even exceeded pre-bleach levels. The translation-to-translocation half-life of NompC, as calculated from the fluorescence recovery in fixed antennae, was ∼3 hr (time constant, τ ∼ 4 hr).

Most notably here, the projected post-recovery (*asymptotic*) fluorescence intensities were higher than their pre-bleach levels (∼40% higher, see Figure 3C). We were wondering if this increase in NompC expression was related to the drastically reduced, effectively eliminated, antennal mobility in agar-embedded flies. If a block of antennal motion (and thus a lack of JO stimulation) leads to an upregulation of transducer channels, then this could be reflective of an underlying activity-dependent control of NompC expression.

### Activity-dependent transcriptional control of NompC

**Figure 4:**
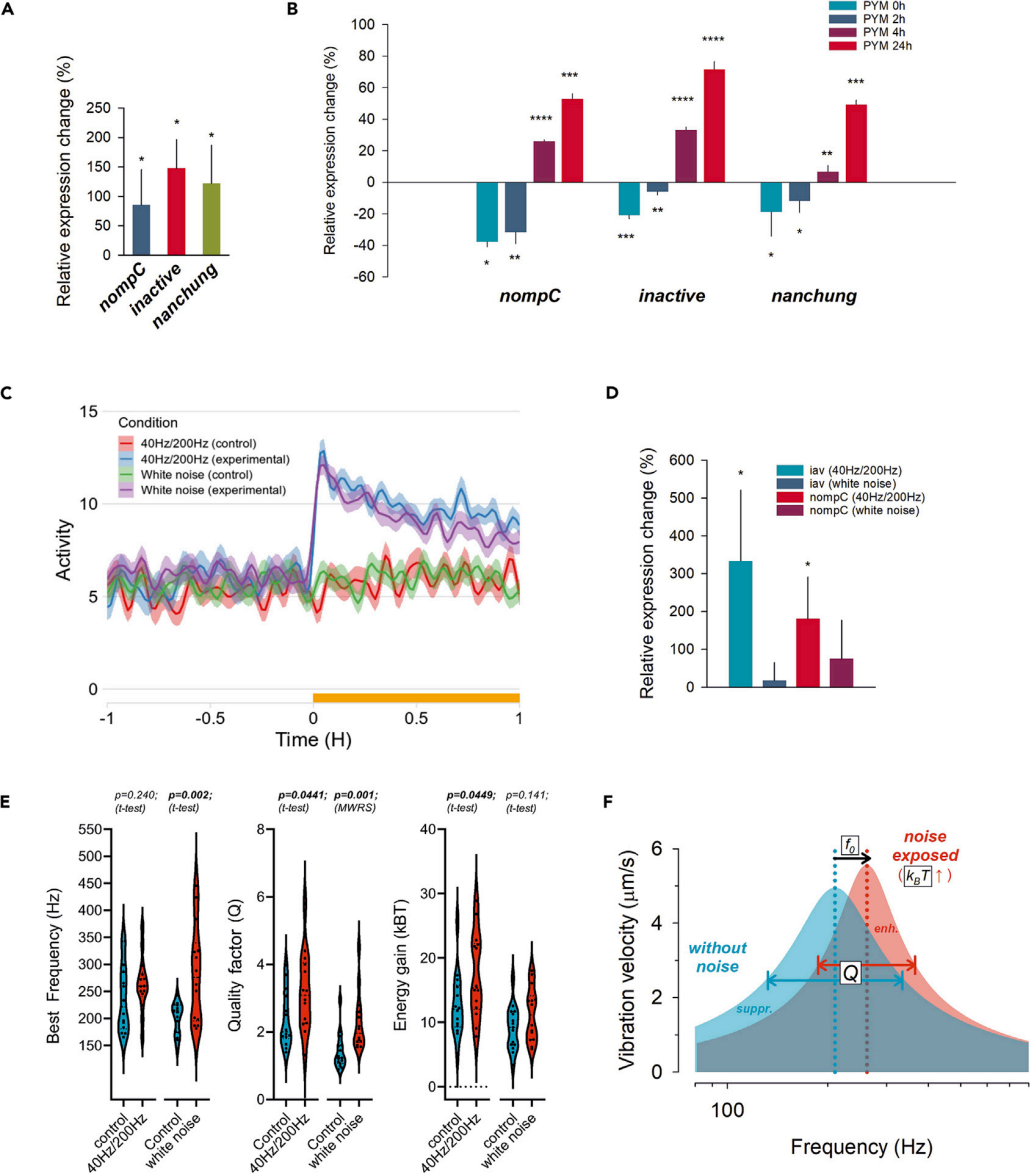
Activity-dependent control of *nompC*expression (A) qPCR analysis showing relative expression of mechanotransduction genes *nompC*, *nanchung* and *inactive* in antennae blocked for 8 hr relative to their freely moving contralateral controls [three negative controls per target, three replicates for each sample and each target]. (B) qPCR analysis of mechanotransduction genes at 4 timepoints after a 1 hr-long Pymetrozine exposure: immediately after exposure (PYM 0h), 2 hr after (PYM 2h), 4 hr after (PYM 4h) and 24 hr after (PYM 24 hr). All qPCR relative expression levels were normalized to endogenous control (*RpL32*) Ct values and finally against control samples. Relative quantitation (2-ΔΔCt) averages and standard deviations were calculated from technical replicates. p values are derived from t-tests of Ct values where ∗p<0.05, ∗∗p<0.01, ∗∗∗p<0.001 and ∗∗∗∗p<0.0001 [three negative controls per target, three replicates for each sample and each target]. (C) Locomotor activity profiles in response to individual stimulus sequences (40/200Hz and white noise, respectively). Stimulus sequences were played in loop for 48 hr[see FigureS5 for more details]. (D) qPCR analysis of mechanotransduction genes after 48 hr long vibrational stimulation (40/200Hz and white noise). All qPCR relative expression levels were normalized to endogenous control (*RpL32*) Ct values and finally against control samples. Relative quantitation (2-ΔΔCt) averages and standard deviations were calculated from technical replicates. pvalues are derived from t-tests of Ct values where ∗p<0.05, ∗∗p<0.01, ∗∗∗p<0.001 and ∗∗∗∗p<0.0001 [five biological replicates and three technical replicates for each target]. (E) Changes of three key parameters of auditory function (left: best frequency; middle: frequency selectivity Q; right: energy gain) after 48 hr exposure to noise (40/200Hz or white noise). (F) Schematic depiction of auditory adjustments to noise in the *Drosophila* antennal ear. Noise exposure leads to shifts in frequency tuning and increased frequency selectivity. The observed shifts, which suppress low (noise) frequencies and enhance (biologically relevant) higher frequencies, can associate with increased energy expenditure.

We used the quantitative polymerase chain reaction (qPCR) to test this hypothesis in three separate experiments that were designed to modulate the mechanosensory input of the fly's antennal ear. As a first proof-of-principal experiment, we tried to block the ears' mechanical input by gluing one antenna while leaving the other free. After 8 hr the second antennal segments of both antennae were dissected and processed for qPCR analysis. Comparing the expression levels of *nompC* between blocked and free antennae revealed a significant (p = 0.04996; ttest) ∼85% increase of expression in the blocked condition (Figure 4A). Interestingly, two other TRP channels, Nanchung (Nan) and Inactive (Iav) (Gong et al., 2004; Nesterov et al., 2015)–which were previously found to be essential for mechanically evoked CAP responses from JO–also showed significant increases (∼122% for Nanchung(p = 0.0487; ttest) and ∼148% for Inactive (p = 0.0159; ttest) after blockage of antennal motion, suggesting activity-dependent expression control mechanisms for key mechanosensory ion channels.

However, with various neuronal populations, with differential response properties (Kamikouchi et al., 2006) and at least two types of transducer channels (sensitive and insensitive ones, ref[Effertz et al., 2012]) present in JO, the initial signaling response to our first experiment is likely to be complex. We thus chose a second approach using the (non-lethal) insecticide Pymetrozine, which specifically targets chordotonal organs (Ausborn et al., 2005). Pymetrozine's molecular target is the heterodimeric ion channel formed by the two interdependent TRPV channels Nanchung (Nan) and Inactive (Iav) (Gong et al., 2004; Nesterov et al., 2015). Functionally, Pymetrozine acts as an agonist, irreversibly opening the Nan/Iav channel, rendering it insensitive to further stimulation. Previous studies showed that the initial strong Ca^2+^ response ceases after a while (Kay et al., 2016); chordotonal neurons eventually become silent and unresponsive to mechanical stimulation (Ausborn et al., 2005; Nesterov et al., 2015) or a second Pymetrozine application (Kay et al., 2016). Notably though, chordotonal neurons do not die and still remain responsive to electrical stimulation (Warren and Matheson, 2018).

Next to *nompC*, we again quantified the post-exposure expression levels of *nanchung* and *inactive*, the direct targets of Pymetrozine. For the experiments, Canton S flies were exposed to food containing 1,000 ppm Pymetrozine for 1 hr and their second antennal segments were dissected immediately afterwards, providing a first timepoint at 0 hr, followed by further timepoints at 2 hr, 4 hr, and 24 hr. The circadian state of the animal was hereby controlled, such that the time of qPCR always fell to the same circadian timepoint. All Pymetrozine exposed flies showed the reported hallmarks of proprioceptive dysfunction, mainly reflected by the incapacity to fly and a severely reduced capacity to climb. The qPCR analysis revealed a highly similar expression pattern for all three TRP channels.

Immediately after exposure (timepoints 0 hr and 2 hr), the expression levels of *nompC*, *inactive*, and *nanchung* decreased, compared to their corresponding controls. At timepoint 0 hr, *nompC* expression decreased by ∼38% (p = 0.0103; ttest) and *inactive* and *nanchung* expression by ∼21% (p = 0.0023; ttest) and 19% (p = 0.0196; ttest) respectively. At timepoint 2 hr, expression levels had slightly risen but were still significantly below control values, with *nompC* expression levels decreased by ∼32% (p = 0.0023; ttest) and *inactive and nanchung* levels by ∼6% (p = 0.0017; ttest) and ∼12% (p = 0.0361; ttest), respectively (Figure 4B). In a second phase (timepoints 4 hr and 24 hr), the expression levels were significantly higher than those of their respective controls, showing relative expression increases of ∼26% (*nompC*, p = 0.00004; ttest), ∼33% (*inactive*, p = 0.00001; ttest) and ∼7% (*nanchung*, p= 0.003517; ttest) four hours post-exposure. At the 24-hr timepoint relative expression had risen further to ∼53% (*nompC*, p = 0.0001; ttest), 72% (*inactive*, p = 0.00002; ttest) and 49% (*nanchung*, p = 0.0002; ttest), respectively.

In a third experimental series, we tested more directly how vibrational activation affects the mechanical response properties of the *Drosophila* antennal ear. To this end, we exposed flies for 48 hr to two different low-intensity vibrational regimes, which we knew to elicit behavioral responses that depend on the antennal ears. Both the combined 40/200Hz and the white noise stimulus increased the flies' locomotor activity (Figures 4C and S5). Whole animal qPCR tests revealed significant increases in *nompC* and *iav* transcript levels post-vibration in the 40/200Hz cohort but not in the white noise cohort (Figure 4D). When quantifying auditory transducer function in free-fluctuation recordings (Figure 4E), we observed a significant increase in frequency selectivity in both 40/200Hz (p = 0.044, ttest) and white noise cohort (p = 0.001, ttest) (Figure 4E, middle). This increase in tuning sharpness was accompanied by a significant increase in energy injection in the 40/200Hz (p = 0.0449, ttest) but not in the white noise cohort (p = 0.141, ttest) (Figure 4E, right). Antennal best frequencies, in turn, were significantly increased in the white noise (p = 0.002, ttest), but not in the 40/200Hz cohort (p = 0.240, ttest) (Figure 4E, left).

## Discussion

The fact that an adult-specific expression of NompC can rescue a functionally deficient NompC mutant background resonates with previous findings (Askew et al., 2015), which showed that transfection of mutant mouse pups with the mammalian candidate auditory channels TMC1 and TMC2 leads to a partial functional recovery of hearing in adult mice. While Askew et al. (2015) clearly demonstrated the feasibility of a transducer-channel based gene therapy, the fact that their approach succeeded also strongly suggests the existence of an underlying homeostatic machinery, which can be exploited to deliver newly synthesized transducer channels to their functional cellular sites. We here explored this question in the model system *Drosophila* using the directly gated mechanotransducer channel NompC as a ‘functional probe’.

(i) the force-transmitting, elastic chain that connects NompC to the antennal receiver will exert a permanent mechanical load on the channels, possibly leading to non-negligible molecular *wear and tear*;
(ii) the extra- and intracellular tethering that is required for the gating of NompC, as well as the transducers' interaction (Nadrowski et al., 2008) with serially connected motor proteins (likely to be dyneins [Karak et al., 2015]) pose a considerable challenge for the continual replacement of functional transducer modules;
(iii) the mechanical coupling between sound receiver and transducers means that changes in the numbers, or molecular properties, of NompC channels can be inferred *in vivo* from the antennal mechanics of intact flies. So far, NompC is the only auditory transducer channel for which this has been established.

It is thus literally the mechanics of their operation which generates both the scientific intrigue of, and the tools for, the analysis of auditory transducer homeostasis.

We here show for *Drosophila* (1) that NompC transducers transcribed *de novo* in the ears of adult flies are being translated, transported, and functionally integrated into their native sites; (2) that NompC-dependent auditory transducers undergo a continual turnover with a logistic time constant of ∼2.3 hr (and a corresponding ∼76% turnover time of ∼9.1 hr), and (3) that the transcription of *nompC* is under activity-dependent control.

Ion channels have long been found to display higher turnover rates than other catalytically active proteins (Sukharev and Sachs, 2012). Reported half-lives for exchangers of mono- or divalent cations, for example, are 2-8 hr, those of cardiac gap junction proteins (e.g., Cx43) even shorter (1-3 hr) (Chen-Izu et al., 2015). Such fast turnover rates, as also found here for NompC, mean that the vast majority of channels are being replaced in the course of any given day. The dynamic regulation of membrane conductances, which takes place on transcriptional, translational, and post-translational levels, is a key feature across the entire nervous system. It has been linked to self-tuning mechanisms, which stabilize neuronal excitation thresholds around cell-specific target activity ranges for input stimuli (Marder and Prinz, 2002).

Our findings suggest a similar stabilization for the NompC-dependent neurons of JO, and possibly chordotonal organs more widely. In response to varying levels of activation, the levels of transducer genes, such as *nompC*, *nan*, or *iav* may move up or down (see Figures 4A,4B, and 4D). The underlying activity-feedback loops appear to be part of an automatic gain control system, which recalibrates JO sensitivity to stabilize, or rebalance, the sensory output (and thus the mean *input* to downstream circuits). Our preliminary findings also indicate a spectral complexity of this process, with different forms of sensory activation leading to different transcriptional and functional responses. These responses partly appear to expend metabolic energy to shift the spectral range of the JO's responsiveness, possibly reflecting an active noise-evasion strategy (Figure 4F). An intriguing question here will be to see what the specific spectral and intensity thresholds of this gain control system are, how they are sensed molecularly; and if–and if so how–they differ between the five frequency-specific subgroups of JO neurons. Seminal work conducted on voltage-gated sodium channels (Baines and Lin, 2018) will provide valuable molecular leads for this line of research. In particular, the roles of action potential firing and its homeostatic regulator *pumilio* (Mee et al., 2004; Muraro et al., 2008) will be worth exploring.

The short turnover times of NompC transducers, as suggested by our FRAP analyses, is also consistent with the rapid transcriptional response we observed after pharmacological manipulation of TRPV-mediated currents in JO neurons. Already at time 0 hr, i.e., maximally one hour after Pymetrozine exposure, a clear downregulation of *nompC* transcription was observed (Figure 4B). The molecular dynamics of the auditory transducer channel NompC, as well as those of the co-expressed channels Nan and Iav, are remarkable, even within the established context of neuronal conductance control. In contrast to many other membrane channels, the loss of which can (at least partly) be compensated by other ion channels (O'Leary, 2018), there appears to be no degeneracy in the auditory transduction chain. A loss of NompC leads to a loss of sensitive transducer gating (Effertz et al., 2012) and sound sensitivity (Effertz et al., 2011); a loss of either Nan or Iav leads to a complete loss of mechanically-evoked conductances in JO neurons (Gong et al., 2004). Any change in the density of these auditory TRP channels is thus likely to affect auditory performance, which would suggest a tight regulation. Together with previous molecular inventories of *Drosophila* hearing (Senthilan et al., 2012) and auditory homeostasis (Keder et al., 2020) these settings form an ideal, low-redundancy model of sensory homeostasis.

NompC has been shown to be part of the primary mechanotransduction pathway in JO (Effertz et al., 2012), i.e., it is mechanically coupled to the external receiver via a series of elastic components, which together form the ‘gating spring’ to which it contributes (Howard and Bechstedt, 2004; Zhang et al., 2015). Its removal and replacement can thus be expected to pose considerable challenges to the underlying homeostatic machinery. To make matters even more precarious, auditory transducer channels are thought to operate at very low copy numbers. Vertebrate inner ear hair cells are thought to have only two transducer channels per stereocilium (Maoileidigh and Ricci, 2019); with ∼30 stereocilia for low-frequency hair cells to ∼300 stereocilia for high-frequency hair cells (Rzadzinska et al., 2004); this corresponds to a total of 60-600 transducer channels per cell. Gating analyses of the fly's inner ear suggest ∼100-1,000 auditory (i.e., ‘sensitive’ as per ref(Effertz et al., 2012)) transducer units per JO; assuming ∼100 neurons contributing to the sensitive transducer gating (JO population A + B[Kamikouchi et al., 2006]); this would correspond to only ∼ 1-10 transducer units per cell. Even if transducer units form multimeric complexes, as has been suggested for many TRP channels, they will still operate at very low numbers and their continual replacement will have to be carefully balanced with the needs of functional continuity. One possibility here could be that homeostatic repair mechanisms are under circadian control and cluster around phases of inactivity. For the mammalian auditory system, a circadian pattern of homeostasis and vulnerability have been suggested (Basinou et al., 2017). Aware of these relations, we made a specific effort to rule out circadian distortions in our dataset. Future studies will have to address the role of the clock in *Drosophila* hearing.

Given the low numbers of active transducer channels, one could also argue that the strong fluorescence of NompC-L-GFP observable in JO cilia without antibody reinforcement is unlikely to result from only <10 GFP molecules but might rather reflect a storage buffer of ready-to-insert NompC transducers, analogous to the readily releasable pools of synaptic neurotransmitters (Kaeser and Regehr, 2017) or the Large Dense-Core Vesicles (LDCVs) that are thought to deliver receptors and signaling molecules to the neuronal membrane (Zhao et al., 2011). Whether such post-translational pools exist and, if so, how they are transported across the cilium and integrated with the existing transcriptional control loops will be one of the key questions of future research into transducer homeostasis. Finally, it is tempting to speculate that a breakdown of homeostasis is the proximate cause for multiple forms of age-related functional decline, such as age-related hearing loss (Gates and Mills, 2005; Keder et al., 2020). The turnover and activity-dependent transcriptional control of the *Drosophila* auditory transducer channel NompC reported here will help to address these questions.

### Limitations of the study

The current study can show that (1) NompC continues to be transcribed, transported, and integrated into functional auditory transducer channels in the adult *Drosophila* ear and that this continual molecular renewal forms part of an activity-dependent readjustment of auditory sensitivity. Although providing some preliminary evidence in this regard, the study cannot show to what extent the molecular recycling also extends to other mechanosensory proteins. Although the study does show that stimuli with different spectro-temporal composition affect NompC transcription in different ways, the sensory logic and molecular pathways of this activity-dependent transcriptional control remain unknown.

## Resource availability

### Lead contact

Joerg T Albert, Professor of Sensory Biology & Biophysics, Ear Institute, University College London, 332 Gray's Inn Road, London WC1X 8EE, UK (email: joerg.albert@ucl.ac.uk)

### Materials availability

No materials were newly generated for this paper. Fly lines used were generated from publicly available lines (Bloomington Drosophila Stock Center; https://bdsc.indiana.edu/).

### Data and code availability

The equations and computational logic used to analyse the data are fully listed in the methods section. All further data can be requested from the corresponding author.

## Methods

All methods can be found in the accompanying transparent methods supplemental file.

## Supplemental information

Document S1. Transparent methods, Figures S1–S5, and Table S1
