# Supplementary material for: Turnover and activity-dependent transcriptional control of NompC in the Drosophila ear
Source: iScience. 2021 Apr 29;24(5):102486. doi: 10.1016/j.isci.2021.102486 (PMC8134069; doi:10.1016/j.isci.2021.102486)
Supplement: Document S1. Transparent methods, Figures S1–S5, and Table S1 [file mmc1.pdf]

iScience, Volume *24*

Supplemental information

Turnover and activity-dependent transcriptional control of NompC in the *Drosophila* ear

Nicholas Boyd-Gibbins, Camille H. Tardieu, Modesta Blunskyte, Nerissa Kirkwood, Jason Somers, and Joerg T. Albert

I. Supplemental Figures

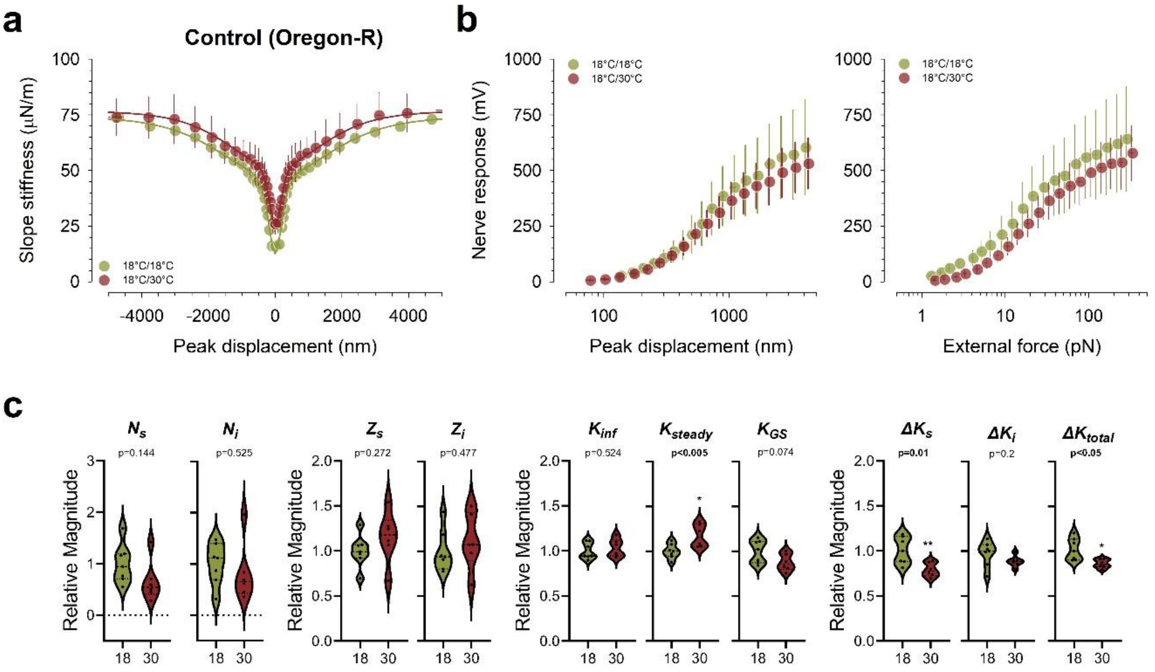

Supplemental Figure 1 (related to Figure 2). Comparison of auditory transduction in [18°C /18°C] and [18°C /30°C] wildtype (Oregon-R) control flies. (a) Slope stiffness of the antennal receivers of control flies (Oregon-R) raised and kept at 18°C (18°C /18°C) (green, N=7) and flies raised at 18°C and transferred to 30°C upon eclosion (18°C /30°C) (red, N=7) as a function of peak displacement. Lines show fits of a two transducer-type gating spring model. Error bars are standard errors of the median. **(b)** Magnitude of Compound Action Potential (CAP) responses as a function of antennal peak displacement (left) and size of the force step (right) for 18°C /18°C and 18°C /30°C Oregon-R control flies. **(c)** Key parameters of auditory transducer function as resulting from the fits in (a). All parameters are expressed in relative terms (divided by their respective values in the 18°C /18°C controls). Parameters (see methods and ref. (Effertz et al., 2012)): number of sensitive (N_s) and insensitive (N_i) transducer channels; sensitive (z_s) and insensitive (z_i) single channel gating forces; asymptotic stiffness (K_{inf}); parallel stiffness (K_{steady}); gating spring stiffness (K_{GS}); stiffness relief for sensitive (ΔK_s) and insensitive (ΔK_i) transducers and both combined (ΔK_{total}).

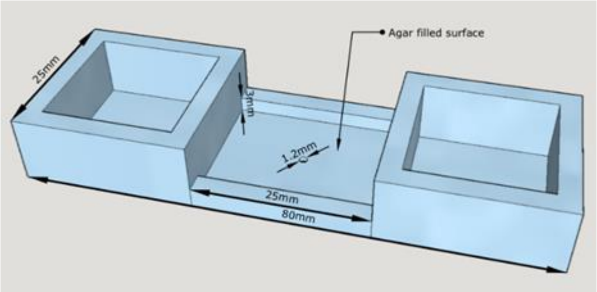

Supplemental Figure 2 (related to Figures 2 and 3). Mount for Fluorescence Recovery After Photobleaching (FRAP) experiments. The design drawings used to produce transparent acrylic fly mounts using a robotic milling machine. The two chambers at either end were used to contain ice to cool the fly and prevent movement. The central chamber was filled with agar. A 50µl pipette tip was inserted into the 1.2mm hole to maintain an opening while the agar set. Flies were mounted into this hole head down and fixed in position with agar cooled to around 25-30°C. Temperatures during the experiments were monitored with a ThermoProbe and kept around 25°C.

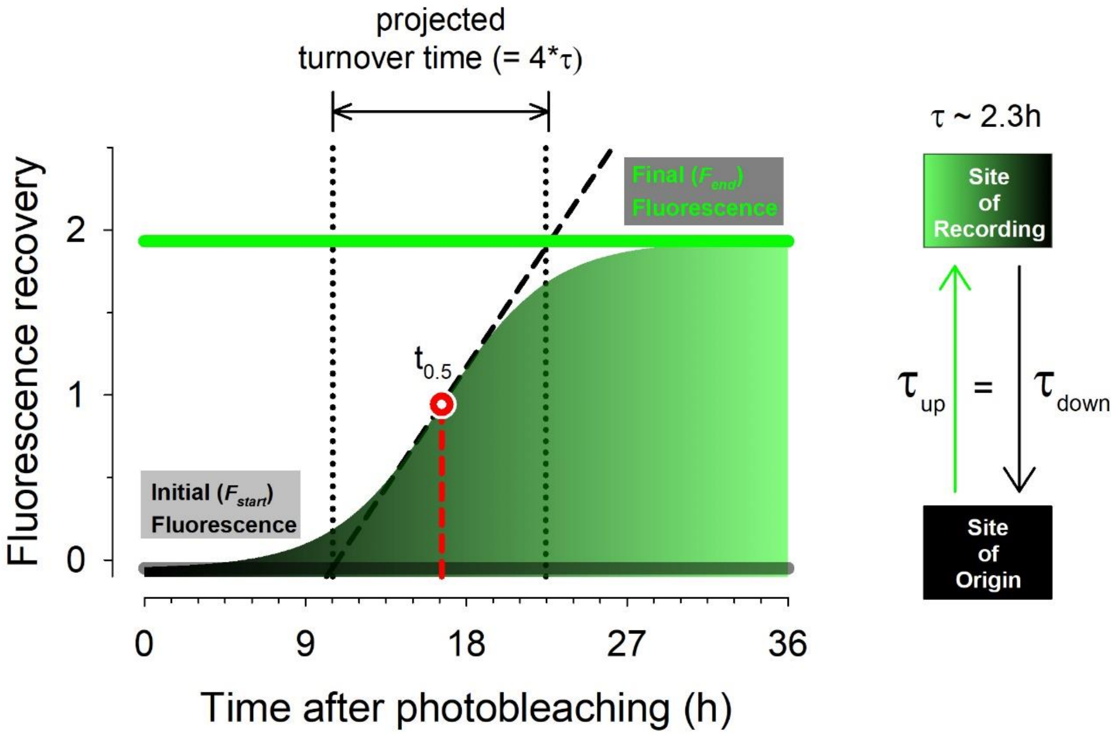

Supplemental Figure 3 (related to Figure 3). Modelling transducer turnover through Fluorescence Recovery After Photobleaching (FRAP). A sigmoidal model was fitted to the FRAP data. The inherently sigmoidal nature of the fluorescence recovery probably resulted from the fact that the effectively photobleached volume inevitably extended beyond the recording sites at the ciliary tips in which NompC is functional and the NompC-GFP signal

most concentrated. The re-emergence of fluorescent NompC thus (at least partially) required the transport (and translation) of new NompC-L-GFP proteins to the ciliary recording sites. This manifested as a time lag in the fluorescence recovery traces. Irrespective of the underlying cause of the delayed onset, however, a sigmoid function, which follows the photo recovery from an initial starting fluorescence to its final asymptotic end value, is a versatile and powerful approach to quantify the kinetics of the recovery process, and thus also the kinetics of the underlying turnover machinery. A minimal assumption model of ion channel homeostasis (and fluorescence recovery by deduction) would posit that - at dynamic equilibrium – a constant number of transducer channels signifies that the removal of old and the addition of new ion channels cancel each other out, leading to a constant fluorescence intensity (F_{end}). After photobleaching the level of fluorescence will drop to zero (if complete) or an arbitrary value below F_{end}. Depending on the noise level in the background and the extent of the bleached area (which – as stated above – might introduce transport-related delays in the fluorescence recovery trace), time lags may occur, which can be absorbed by a shift of the midpoint parameter $t_{0.5}$ (see red dotted circle and red dashed line). In all cases, the (unchanged) kinetics of the underlying turnover process will be faithfully captured within the time constant parameter (τ). The turnover time (which is calculated as $4*\tau$), finally, provides a useful estimate for the time it takes to recover the majority of bleached ion channels (corresponding to ~76% of the final, asymptotic fluorescence intensity). The here chosen definition of turnover time is the time it would take the process to move from F_{start} to F_{end} at the highest observable speed (i.e. along the line of maximum slope, see dashed line in figure). This simple equilibrium model assumes an identical time constant τ for the assembling and disassembling process (τ_{up} and τ_{down}, respectively).

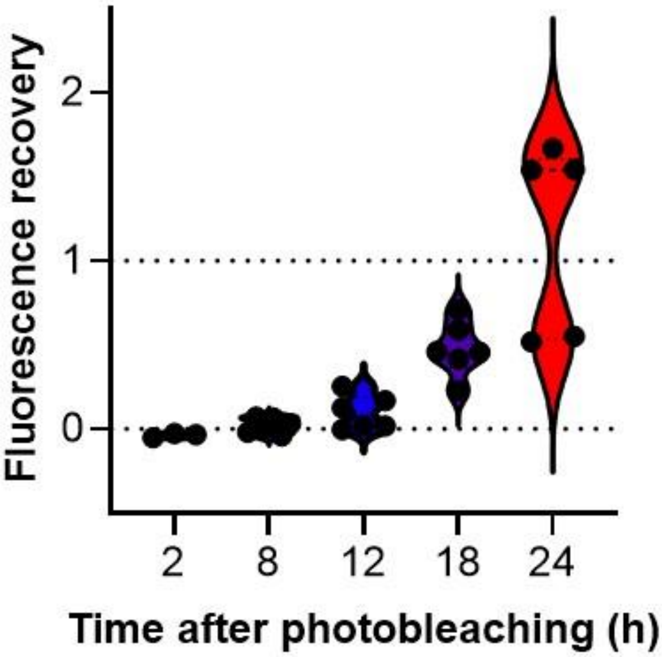

Supplemental Figure 4 (related to Figure 3). Plot showing individual data points for the box plots of Figure 3c.

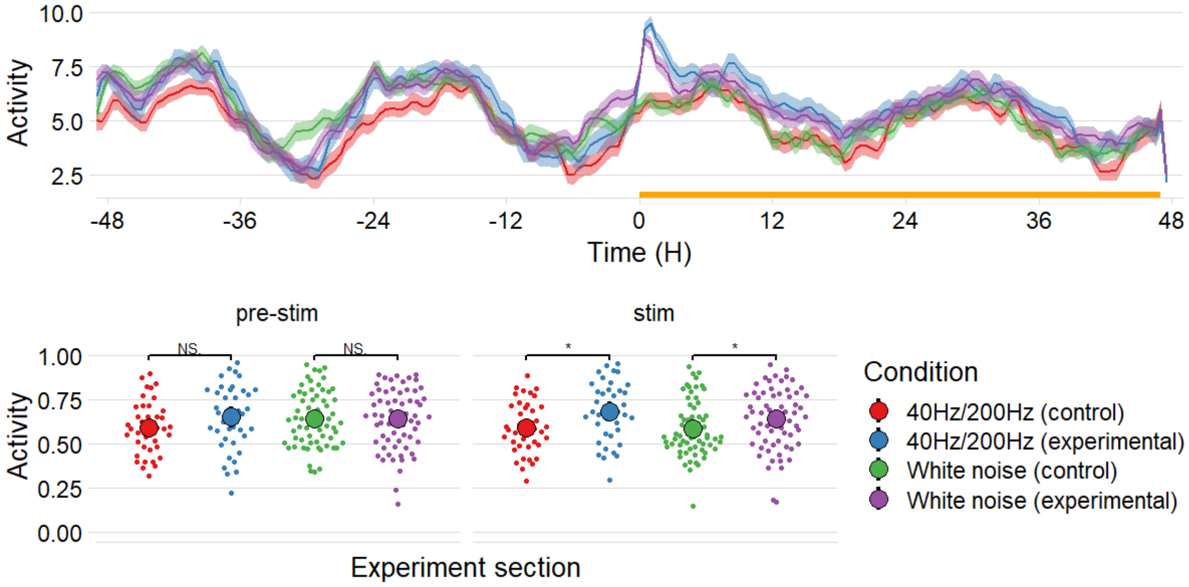

Supplemental Figure 5 (related to Figure 4). Plot showing locomotor activities before and during the 48h-long vibrational activation. (**top**) Average activity for each group across the experiment 48hours prior to stimulus onset to 48hours after stimulus onset (yellow bar indicates stimulus period). A one-hour low pass filter is first applied to the raw activity counts to remove high frequency noise and data is binned at 30-minute intervals. (**bottom**) Average

activity for each individual fly across the 48hours pre-stimulation (pre-stim) and of the 48hours of stimulation (stim). No significant difference between average activity is observed in the two stimulation types *prior to stimulus onset.* Significant difference between average activity is observed between both stimulation type control and experimental groups, $p = 0.011$ and $p = 0.041$ for 40/200Hz and white noise respectively, Wilcoxon Rank Sum test.

Supplemental Table 1 (related to Figure 3). Plot showing fit parameters for sigmoid recovery fits (including lower and upper confidence intervals). N=5

Parameter	Value	Lower CI (95%)	Upper CI (95%)
$t_{0.5}$ (h)	1.567e+1	1.593e+1	1.549e+1
F_{start}	2.118e-1	1.171e-1	3.033e-1
F_{end}	1.435e+0	1.101e+0	1.766e+0
τ *(h)*	2.287e+0	2.155e+0	2.375e+0

II. Transparent Methods

Fly lines and temperature regimes

All flies were raised on standard media at 70% humidity with a 12 hr:12 hr light:dark cycle. For temperature-controlled conditional gene expression experiments (using $Gal80^{ts}$) flies were kept at either 18°C or 30°C. For FRAP experiments flies were kept at 25°C. Virgin males and females were isolated on the day of eclosion using CO_2 sedation for subsequent crossing or experiments. All electrophysiological and biomechanical experiments were conducted at room temperature (18-20°C). Food was changed weekly and vials were populated with a maximum of 10 animals each.

To obtain *nompC* null mutant background fly lines with NompC rescue under temperature control the homemade balanced line carrying the second chromosome deficiency *Df(2L)cl^{h2}* and the temperature-sensitive Gal4 inhibitor $TubGal80^{ts}$ (*Df(2L)cl^{h2}/Cy*; $tubGal80^{ts}$ /MKRS) was crossed to the homemade balanced line carrying the mutant *nompC^{3}* allele (Walker et al., 2000), a *UAS-nompC-L-GFP* construct, and the nompC-Gal4 driver (Cheng et al., 2010) (*nompC^{3}*,*UAS-nompC-L-GFP/CyO*;*nompC-Gal4/MKRS*).

For FRAP experiments, balanced lines were obtained by crossing homemade lines to double balancer line y w; *Sp*/*Cy*;MKRS/TM6b in order to create the line nompC^3, *UAS-nompC-L-GFP*/*Cy* ; NP0761/*MKRS*. NP0761 flies are reported in Kamikouchi *et al.*, 2016 (Kamikouchi et al., 2006) and *UAS-NompC-L-GFP* in Chen *et al.*, 2010 (Cheng et al., 2010). Virgin males were collected after eclosion and aged until day 10 on normal food in a 25°C incubator submitted to 12 hour dark and 12 hour light cycles and 70% humidity. Pymetrozine experiments were conducted on 6-10 day old Canton-S males.

Biophysical and electrophysiological measurements

For analysis of JO function, flies were mounted as described previously (Albert et al., 2007). Briefly, flies were attached ventrum-down to the tip of a Teflon rod using blue light cured dental glue. All potentially moving parts of the animal were secured including all legs, wing tips, and the back of the head to the upper thorax. The non-experimental (left) antenna was glued to the head to prevent any sound-induced movements or electrical signalling. The first and second antennal segments of the experimental (right) antenna were secured with glue to the head and a bridge of glue was made to the left antenna thereby anchoring the experimental antenna and ensuring all movements of the antenna were due to the experimental stimuli. The Teflon rod with the fly attached at the top was mounted in a micromanipulator that allowed accurate positioning of the fly for the experiment.

All experiments were carried out on an active vibration isolation table (model 63-564; TMC, USA). The arista of the experimental antenna was positioned perpendicular to the beam of a laser doppler vibrometer (PSV-400; Polytec, Germany) with an OFV-70 close-up unit (70 mm focal length) and a DD-500 displacement decoder. The laser beam was focused on the tip of the arista.

Two electrostatic actuators were placed in push-pull mode on either side of the arista, with one actuator ~150µm in front and one ~150µm behind the arista. A tungsten electrode (charging electrode) was inserted into the fly's thorax and used to raise the animal's electrostatic potential to ~-20V against ground. Electrostatic actuation (EA) of the antenna achieved by this setup is described in detail in refs (Albert et al., 2007) and (Effertz et al., 2012).

A second, electrolytically sharpened tungsten electrode (recording electrode) was inserted into the fly's head close to the base of the experimental antenna in order to make recordings of stimulus-evoked compound action potentials (CAPs) from the antennal nerve. The charging electrode also served as a reference electrode for the recording electrode.

Electrostatic force step stimulation of the antenna

At the start of each experiment, the size of the maximum command voltage (and resulting force step) was calibrated so that it produced a steady-state displacement of ~ ±8µm. For the force step experiments, 25ms-long step stimuli, with incrementally smaller steps, were played to the experimental antenna and the displacement responses were digitized at a rate of 100 kHz using a CED Power 1401 mk II A/D converter. Signals were loaded, and analysed, in the Spike 2 software (both Cambridge Electronic Design Ltd., Cambridge, England). The step programme consisted of 20 steps of exponentially smaller size, each step resulting in a final smallest force step of around ~1-1.5 pN magnitude. During the experiment the applied stimulus voltage and stimulus-coupled nerve responses were also recorded.

Data was analysed as described previously (Albert et al., 2007, Weinberger et al., 2017). Briefly, *Drosophila* antennae respond to force step stimuli with an initial displacement overshoot (X_{peak}) followed by a recoil after which a steady state displacement (X_{steady}) is reached. From the antennal displacement response the applied force, *F*, can be calculated from the maximum acceleration, $\ddot{X}_{onset}$, at the onset of forcing (the mass of the system is assumed to be $m = 5 \times 10^{-12} g$):

$$F = m\ddot{X}_{onset}$$

To work out the steady state stiffness (K_{steady}) the steady state displacement (X_{steady}) was found by fitting an exponential to the adaptation component of the displacement response and taking X_{steady} to equal the asymptotic value. This is then used as below:

$$K_{steady} = \frac{d(m\ddot{X}_{onset})}{dX_{steady}}$$

At the displacement peak, X_{peak} , the dynamic stiffness of the receiver can be found from:

$$K_{peak} = \frac{d(m\ddot{X}_{onset} - m\ddot{X}_{peak})}{dX_{peak}}$$

In this calculation, the effective force experienced by the receiver, $m\ddot{X}_{onset}$, takes into account the inertial forces resulting from the receiver's (non-zero) mass, $m\ddot{X}_{peak}$.

In further analyses, values for the parallel stiffness K_{par}, the gating spring stiffness, K_{GS}, and the asymptotic stiffness, K_{∞}, are determined by fitting a two transducer-type gating spring model (described below) to the force/displacement data (see (Effertz et al., 2012) for details). K_{par} is equal to K_{steady} if adaptation is complete, which has been shown for the ears of wildtype flies (Albert et al., 2007). K_{GS}, the total gating spring stiffness, is then simply calculated from $K_{\infty} - K_{steady} = K_{GS}$.

Fits of a two-transducer gating spring model

A symmetric gating spring model that includes two opposing populations of transducer complexes is fitted to the stiffness data obtained from the above described force step stimulation experiments. The gating spring model applied here is identical to the one described in (Effertz et al., 2012). Below is a summary of the model.

For each transducer channel population, the open probability is defined as:

$$p_o(X) = \frac{1}{1 + e^{-\frac{z(X - X_0)}{k_B T}}}$$

In this expression, *z* is the change in force in a single gating spring as the channel is opened, X_0 is the displacement of the antenna at which the open probability is 0.5, k_B is the Boltzmann constant and *T* is the absolute temperature. This is simplified by the assumption that $X_0 = 0$, giving:

$$p_o(X) = \frac{1}{1 + e^{-\frac{zX}{k_B T}}}$$

In the model used here it is assumed that two opposing populations of transducers have inversely related open probabilities. Hence, as the open probability of one population increases, the open probability of an opposing population decreases. As the gates of one population open, the other population's gates will close.

$$K(X) = K_{\infty} - \left(\frac{N_s z_s^2}{k_B T}\right) p_{o_s}\left(1 - p_{o_s}\right) - \left(\frac{N_i z_i^2}{k_B T}\right) p_{o_i}\left(1 - p_{o_i}\right)$$

K_{∞} is here the asymptotic stiffness of the system, the stiffness of the receiver for very large displacements when all transducers are either fully open or fully closed; p_{o_s} is the open

probability of the population of sensitive transducers and p_{o_i} is the open probability of the insensitive transducers; N_s refers to the number of sensitive transducers and N_i to the number of insensitive transducers; z_s and z_i describe the respective single channel gating forces for the two transducer types.

Extent of nonlinearity

The extent of nonlinearity in the antenna's response was determined as previously described by Albert et al, 2007 (Albert et al., 2007).

As described above, the stiffness of the saturated system, K_∞, is reduced by the contributions of two populations of transducers and depends on the single channel gating force, *z*, of each type of transducer, the number of channels, *N*, and the open probability p_0. For open probabilities of 0.5, the stiffness relief, ΔK_S, of the system is maximum and can be written as

$$\frac{Nz^2}{4k_BT}$$

Where k_B is the Boltzmann constant (4.11×10^{-21} J) and T the absolute temperature (here 293.15 K). By dividing by the saturated stiffness, K_∞, a ratio is obtained that describes the total extent of nonlinearity in the receiver's response, NL_{total}. This provides a ratio of the reduction in stiffness to the total stiffness of the saturated system:

$$NL_{total} = \frac{Nz^2}{(4k_BT \cdot K_\infty)}$$

This total extent of nonlinearity is comprised of both the contribution to nonlinearity of the sensitive transducers and the insensitive transducers summed together, and hence should be written as below:

$$NL_{total} = \frac{N_s z_s^2}{(4k_BT \cdot K_\infty)} + \frac{N_i z_i^2}{(4k_BT \cdot K_\infty)}$$

In this way the total extent of nonlinearity in the system can be calculated from the fit parameters obtained from fitting the gating spring model described above, and the contributions predicted made by the sensitive and insensitive populations of transducers can be also be assessed separately.

While this ratio quantifies a receiver's extent of nonlinearity, changes to its asymptotic stiffness, K_∞, will affect this extent. Comparing the extents of nonlinearity between different flies, or different fly conditions, where K_{inf} changes, may thus be misleading.

To allow for a more direct assessment of the stiffness relief that results from auditory transducer gating, we also calculated the absolute stiffness relief, ΔK, that the two

transducer populations introduce into the receiver mechanics (as per ref. (Hudspeth et al., 2000)). For the sensitive population this stiffness relief is ΔK_s

$$= \frac{N_s z_s^2}{4k_B T}$$

and for the insensitive population this stiffness relief is ΔK_i

$$= \frac{N_i z_i^2}{4k_B T}$$

Leading to a combined stiffness relief of ΔK_{total}

$$= \frac{N_s z_s^2 + N_i z_i^2}{4k_B T}$$

FRAP mount

Agar was chosen as a suitable medium to trap flies during FRAP experiments. The use of agar allowed for minimising the amount of glue necessary to stabilise the flies in a desired position and also kept flies hydrated. An individual (one-fly) mount milled in a transparent cast acrylic plate (see Supplementary Figure 1) featuring dimensions as annotated below was used. A 1.2mm hole housed the fly, head facing down to where cover glass was attached. This mount was designed to be virtually the same size as a typical microscopic slide and suitable for an inverted microscope. It is possible to cool the mount down by inserting ice into the two wells on each side of the mount. This is particularly useful in the case of high temperatures in the confocal chamber.

The tips of 50µl pipettes were used to help shape an opening on the acrylic mount where the agar would be poured to set. One millilitre of 1.5% agar gel prepared with Low Gelling Temperature Agarose (Sigma-Aldrich) was cast in each mount and left to set for 30min. Some liquid agar was kept in a 37°C incubator. One hour before the experiment, the flies were mounted as follows. Flies were anesthetised with CO_2. After 1min on CO_2, the fly was transferred into the agar opening and was carefully pushed up the small agar "tunnel" until its head was slightly above the acrylic side of the mount. At that stage, the head was rapidly fixed into position with dental glue. The glue was cured with a curing light. 20µl of the liquid agar preserved at 37°C and cooled down to around 25-30°C was then pipetted in the agar "tunnel". By solidifying virtually instantly, the agar limited leg movements. Finally, a 22mm x 22mm glass coverslip with glycerol was placed on the head. The coverslip was then immobilised with electrical insulating tape.

Photobleaching and Imaging

FRAP experiments were conducted on a Zeiss LSM 510 confocal microscope. 3µm optical slice sections were acquired for pre-bleach and post-bleach stacks with a Plan-Neofluar 40x/1.3 Oil objective. The argon laser was used at 25% in order to detect GFP proteins with a 245µm pinhole. Saturated pixels were avoided by using a range indicator palette. Photobleaching was triggered and monitored manually in an area of approximately 66 x 44µm targeting the cilia previously selected from the pre-bleach stack. The argon laser power was changed to 100% at which times-series lasted an average of 300 frames for approximately 3min30sec. Once the fluorescence decay speed had reduced to minimal changes, the time-series was stopped, and the post-bleach stack was taken. For each fly, the photobleaching time, rotation of the stack and side of the antenna (right or left antenna) were logged. Between post-bleach imaging (directly after photobleaching) and recovery imaging, the mounts were kept on a plate lined with damp tissue and placed in a 25°C incubator.

Image and data analysis

The software Image J was used to quantify fluorescence intensity in pre-bleach, post-bleach and recovery stacks. On the pre-bleach stack, a rectangular region of interest (ROI) was delimited as similar as possible to the bleached area both in dimensions and in depth. The same procedure was followed for background intensities. Arrays of intensities present in the JO ROI and background ROI throughout the stack were acquired. This was repeated for post-bleach and recovery stacks. Only the fluorescence values corresponding to the bleached slice plus two slices below and two slices above were selected for analysis. These fluorescence intensity values were then calculated and analysed as follows:

$$JO_{postbleach(timepoint)} \,/\, JO_{prebleach(timepoint)}$$

Statistical tests were computed in SigmaPlot (Systat Software, Inc.): a four-parameter sigmoid fit was applied to the fluorescence recovery data points such that see also Supplemental Figure 1:

$$f = \frac{F_{start} - F_{end}}{(1 + e^{\frac{t - t_{0.5}}{\tau}})} + F_{end}$$

With t being the time of measurement, F_{start} being the relative fluorescence at *t*=0, F_{end} being the asymptotic relative fluorescence at *t*=∞, $t_{0.5}$ being the time at 50% recovery (centre point) and τ being the time constant. Corresponding half-life values were calculated as $\tau * \ln(2)$.

Pymetrozine exposure

Pymetrozine was provided by Syngenta, UK. The powder was prepared and homogenised in MilliQ water as a 1000ppm stock (or 4.6mM). Simple sugar and agar food was prepared with 1% agar and 5% sucrose in water. 60µl of the Pymetrozine solution was applied onto the surface of the food once it had set. The solution was then left to dry overnight. Control or sham food was prepared using the same ingredients cited above without applying Pymetrozine.

Canton-S virgin males were aged 6 to 10 days. They were kept in a 12 hr:12 hr light:dark regime at 25°C. Prior to Pymetrozine exposure, the flies were starved for 1 hour in empty food vials with wet tissue to prevent desiccation. The flies were then transferred onto Pymetrozine or sham food and exposed for one hour.

Exposed flies were then transferred onto typical sugar/yeast food. Four time points were chosen: 0, 2, 4 and 24 hours after Pymetrozine exposure. In order to account for putative circadian fluctuations in mechanotransducer factors' gene expression, controls were dissected at the same four time points and at the same time of the day in a strict one-hour window. In total, 35 flies were dissected for each time point.

Dissection and RNA extraction

Flies were anesthetised on ice and their second antennal segments were dissected in a strict one hour window. Dissection of the second antennal segment involves pulling out the third segment followed by pinching the first and second segment joint with sharp forceps in order to preserve the JO encapsulated in the second antennal segment (a2). They were then collected in 1% β-mercaptoethanol in Lysis Buffer (provided in Qiagen RNeasy Mini Kit). As soon as the dissections were completed for one time point, the samples were frozen at -25°C. After thawing on ice, samples were homogenised with an ultrasonic device (Hielscher UP200H sonicator) set at 100kHz for 20 to 30sec. The sonicator tip was carefully washed with ethanol after each use. RNA was extracted from samples according to the Qiagen RNeasy Mini Kit protocol. RNA samples were then stored at -80°C.

Reverse transcription and pre-amplification

Reverse transcription was carried out with the High Capacity RNA to cDNA kit (Applied Biosystems). RNA samples, Enzyme Mix and Buffer Mix were thawed on ice and briefly vortexed. 10µl Buffer Mix, 1µl Enzyme Mix and 9µl RNA were mixed and briefly centrifuged in

PCR tubes. Thermal cycling was programmed for 60min at 37°C and 5min at 95°C. For immediate use, cDNA was kept at 4°C.

In order to proceed to pre-amplification with TaqMan PreAmp Master Mix Kit, a “pooled assay” of Taqman primers was prepared. TaqMan primers were mixed together and diluted 1:100 in TE buffer. 25µl PreAmp Master mix, 12.5µl “pooled assay” and 12.5µl cDNA were homogenised in PCR tubes. Thermal cycling was programmed 10min at 95°C followed by 14 cycles of 15sec at 95°C and 4min at 60°C. The pre-amplified cDNA was diluted 1:20 in TE buffer and stored at 4°C if the qPCR was performed immediately after or -25°C if the qPCR was performed later.

qPCR

Real time Polymerase Chain Reactions were run on a Step One Plus ABI machine. Prior to the reaction, the 96 well plate set up was designed with the Step One Plus software. Three negative controls were run per target as well as three replicates for each sample and each target. Ribosomal Protein L32 (PRL32) was chosen as the endogenous control and day 1 sample as the control sample. Reactions were prepared in Eppendorf tubes considering the chosen reaction volume per well was 10µl containing 0.5µl TaqMan Gene Expression Assay (primer), 5µl Gene expression Assay Master Mix and 4.5µl cDNA. (See table below for list of TaqMan Gene Expression Assay). The Eppendorf tubes were centrifuged briefly and 10µl of the reaction mix was pipetted in each well in a MicroAmp Fast Optical 96-Well Reaction Plate. The plate was covered with a MicroAmp® Optical Adhesive Film and placed in the Step One Plus machine, programmed for two minutes at 50°C, ten minutes at 95°C followed by 40 cycles of 15 seconds at 95°C and one minute at 60°C.

Table 1. TaqMan Gene Expression Assay.

Gene	*TaqMan probe ID*	*Quencher*
**nompC**	Dm01808271_m1	FAM
**inactive**	Dm01833375_g1	FAM
**nanchung**	Dm01805137_g1	FAM
**RPL32**	Dm02151827_g1	VIC

Cycle threshold (C_t) values were extracted from the Step One Plus Software data analysis. The $\Delta\Delta C_t$ and relative quantitation values were calculated in Excel such that:

$$\Delta C_t = (C_t gene\ x\ control - C_t endogenous\ control)$$

$$\Delta\Delta C_t = \Delta C_t - (C_t gene\ x\ condition - C_t endogenous\ condition)$$

$$RQ = 2^{-\Delta\Delta C_t}$$

The three RQ values were averaged for each triplicate and standard deviations generated in Excel. Statistical tests were performed in Excel.

Protocols used to test activity-dependent transcriptional control and auditory tuning (48h stimulation series)

To test if vibrational stimulation affects NompC transcription levels, and functional properties of the antennal ear, flies were stimulated for 48 hours under different regimes in climate-controlled incubators (Percival Scientific, Inc., USA; 70%RH at 21°C). Individual flies were loaded into DAM5M monitors (TriKinetics Inc, USA) provided with sugar-agarose food, which were monitored on DAMSystem3 software. Activity monitors were attached to a bass loudspeaker, to allow for vibrational stimulation (equivalent to (Simoni et al., 2014)). Experiments were run with two different stimulus groups and two corresponding (silence) control groups. Stimulus patterns: (i) alternating 0.5s long 40Hz/ + 200Hz stimuli, separated by 0.5s silence and (ii) 0.5s long randomized white noise sequences (30-1,000Hz), separated by 0.5s silence. Each group comprised of 32 flies. Stimuli were designed in the Spike2 software and played through a CED Power 1401-3 A/D converter (both Cambridge Electronic Design Ltd, UK). In the 40/200Hz cohort, flies were exposed to the stimulus sequence for one minute, followed by one minute of silence. This was repeated for 48 hours until the end of experiment. Similarly, the white noise cohort were exposed to the white noise stimulus sequence for one minute followed by one minute of silence for 48h. Both stimulus cohorts had individual (silent) controls.

After 48-hour stimulation, flies were either harvested for qPCR or their auditory properties were tested in free fluctuation recordings using a Laser Doppler Vibrometer (LDV). For the free fluctuation recordings, flies were mounted as described above (see section 'Biophysical and electrophysiological measurements'). Mounted flies were left to rest for an hour to allow for recovery from the cold anaesthesia and gluing procedure.

RNA extraction from whole-body samples

A whole-body RNA harvest was performed on flies after 48 hours of exposure to the different stimulus regimes. This was used to probe for transcriptional control of mechanotransducer channels across mechanosensory organs. Flies were collected and cryopreserved in liquid nitrogen after 48-hour stimulation. Flies were homogenised in 1% β-mercaptoethanol in Lysis Buffer (provided by PureLink RNA Mini Kit) using pestle. This was followed by RNA extraction using PureLink RNA mini kit. Samples were stored at -80°C.

Reverse transcription and pre-amplification of whole-body samples

RNA samples and reverse transcription buffers were thawed on ice and briefly centrifuged (supplied by Applied Biosystems). 10X RT Buffer, 25X dNTP Mix, 10X RT Random Primers, MultiScribe Reverse Transcriptase and Nuclease-free water were briefly centrifuged. The reagents were mixed with RNA samples in PCR tubes. The thermal cycling was programmed for 10min at 25°C, 120min for 37°C, 5min for 85°C, and hold at 4°C.

The TaqMan PreAmp Master Mix Kit (provided by Applied Biosystems) was used to increase the concentration of target genes – *nompC* and *iav*. The 0.05X Pooled assay mix was prepared with TaqMan primers and nuclease-free water. Each reaction contained 25μl of 1X TaqMan PreAmp Master Mix, 12.5μl of Pooled assay mix and 12.5μl of cDNA. Thermal cycling was programmed as follows: 10min at 95°C, 10 cycles of 15sec at 95°C and 4min at 60°C, and the last step involved 99°C for 10min and 4°C on hold. The pre-amplified cDNA was diluted 1:100 in nuclease-free water and stored at 4°C for immediate use or at -25°C for later use.

Modification of qPCR tests for activity-dependent transcriptional control (whole body samples, 48h series)

Real Time Polymerase Chain Reactions were run on a Step One Plus ABI machine and conducted as described above. Only three modifications were made: For whole-body samples, (i) five biological replicates and three technical replicates for each target were tested; (ii) silent cohort flies were chosen as control samples; (iii) genes tested were *nompC*, *inactive* with *RpL32* as control gene (see table below).

Table 1. TaqMan Gene Expression Assay.

Gene	**TaqMan Probe ID**	**Quencher**
nompC	Dm01808271_m1	FAM

inactive	Dm01833375_g1	FAM
RpL32	Dm02151827_g1	VIC

References

Albert, J. T., Nadrowski, B. & Göpfert, M. C. 2007. Mechanical signatures of transducer gating in the *Drosophila* ear. *Curr Biol,* 17**,** 1000-6.

Cheng, L. E., Song, W., Looger, L. L., Jan, L. Y. & Jan, Y. N. 2010. The Role of the TRP Channel NompC in Drosophila Larval and Adult Locomotion. *Neuron,* 67**,** 373-380.

Effertz, T., Nadrowski, B., Piepenbrock, D., Albert, J. T. & Gopfert, M. C. 2012. Direct gating and mechanical integrity of Drosophila auditory transducers require TRPN1. *Nature Neuroscience,* 15**,** 1198-U43.

Hudspeth, A. J., Choe, Y., Mehta, A. D. & Martin, P. 2000. Putting ion channels to work: Mechanoelectrical transduction, adaptation, and amplification by hair cells. *Proceedings of the National Academy of Sciences of the United States of America,* 97**,** 11765-11772.

Kamikouchi, A., Shimada, T. & Ito, K. 2006. Comprehensive classification of the auditory sensory projections in the brain of the fruit fly Drosophila melanogaster. *Journal of Comparative Neurology,* 499**,** 317-356.

Simoni, A., Wolfgang, W., Topping, M. P., Kavlie, R. G., Stanewsky, R. & Albert, J. T. 2014. A Mechanosensory Pathway to the *Drosophila* Circadian Clock. *Science,* 343**,** 525-528.

Walker, R. G., Willingham, A. T. & Zuker, C. S. 2000. A Drosophila mechanosensory transduction channel. *Science,* 287**,** 2229-34.

Weinberger, S., Topping, M. P., Yan, J. K., Claeys, A., De Geest, N., Ozbay, D., Hassan, T., He, X. L., Albert, J. T., Hassan, B. A. & Ramaekers, A. 2017. Evolutionary changes in transcription factor coding sequence quantitatively alter sensory organ development and function. *Elife,* 6.
